# HPV Infection and Prognostic Factors of Tongue Squamous Cell Carcinoma in Different Ethnic Groups from Geographically Closed Cohort in Xinjiang, China

**DOI:** 10.1155/2016/7498706

**Published:** 2016-03-10

**Authors:** Hua Zhang, Yang Zhang, Huarong Zhao, Huerxidan Niyaz, Pan Liu, Lei Zhang, Songan Zhang, Yiming Reheman, Yongxing Bao, Xinhua Chen

**Affiliations:** ^1^First Affiliated Hospital of Xinjiang Medical University, Oncology Center, 137 Liyushan Road, Urumqi 830011, China; ^2^The Department of Hepatobiliary and Pancreatic Surgery, The First Affiliated Hospital, Zhejiang University, 79 Qinchun Road, Hangzhou, Zhejiang 310003, China; ^3^Collaborative Innovation Center for Diagnosis Treatment of Infectious Diseases, 79 Qinchun Road, Hangzhou, Zhejiang 310003, China

## Abstract

*Background.* The effect of HPV infection status and ethnic differences on the prognosis of tongue squamous cell carcinoma in Xinjiang presents an interesting set of conditions that has yet to be studied.* Methods.* A comprehensive analysis of clinical data was undertaken for a cohort consisting of 63 patients with tongue squamous cell carcinoma recruited from three ethnic groups in Xinjiang. PCR was used for the detection of HPV16 and HPV18 infections. Kaplan-Meier survival analysis was used for analyzing survival outcome in addition to the assessment of other prognostic factors.* Results.* The overall infection rate for HPV was 28.6% (18/63); the 5-year survival rate among the HPV-positive patients was 47.8% and 30.3% for HPV-negative patients. The survival rate for HPV-positive patients who received radiotherapy and chemotherapy was better than for those who did not receive radiotherapy and chemotherapy. N staging and HPV infection were found to be two independent and significant prognostic factors.* Conclusion.* HPV-positive patients with tongue squamous cell carcinoma are more sensitive to chemotherapy. Higher N staging indicates poor prognosis.

## 1. Introduction

The incidence of head and neck cancer is ranked sixth amongst malignant tumors and the most common manifestation of this cancer is squamous cell carcinoma. Numerous studies show that human papilloma virus (HPV) plays an important role in the etiology of head and neck squamous cell carcinoma [1, 2]. HPV infection has been reported especially risky for Caucasian patients, sometimes increasing the risk tenfold as compared to other ethnic groups [3]. Some reports also show that HPV-positive patients have better sensitivity to radiotherapy and chemotherapy and thus a better prognosis than HPV-negative patients [4, 5]. However, the extent to which these factors may impact different ethnic groups within Xinjiang is unknown. The Uygur, Kazakh, and Han are the three major ethnic groups in the Xinjiang Uygur Autonomous Region. They reside within a relatively closed and mountainous geographic area but their genetics and dietary preferences are quite different. The effect of HPV infection status and ethnic differences in Xinjiang on tumor prognosis is an interesting question that has yet to be studied. Here we report our findings concerning HPV infection in tongue squamous cell carcinoma among three ethnic groups of Xinjiang while also taking into consideration other prognostic factors.

## 2. Materials and Methods

### 2.1. Clinical Data

#### 2.1.1. General Information

63 tongue squamous cell carcinoma cases were recruited from June 2004 to June 2013 from the First Affiliated Hospital of Xinjiang Medical University. The Ethical Committee of the hospital approved the study plan. Their TNM stage was declared according to guidelines of the International Union against Cancer (UICC) version 2002. The patients receiving surgical treatment were diagnosed by pathological slides of the tumors located on the base of the tongue and the patients with nonsurgical treatment were diagnosed by radiographic examination (CT, MRI, and PET-CT) and biopsy pathology. Human Papillomavirus (HPV) DNA detection was performed on paraffin-embedded tissues. The cohort consisted of 25 males and 38 females, aged 28 to 84 (median age 56 years). The ethnic composition of the patients was 19 Uighur, 10 Kazak, and 34 Han. 19 were smokers and 44 nonsmokers; 27 patients drink frequently and 36 did not. According to UICC/AJCC2002, 36 cases were in stage I, 12 in stage II, 7 in stage III, and 8 in stage IV; the pathological differentiation showed that 40 tumors were well differentiated, 23 moderately differentiated, and zero poorly differentiated.

#### 2.1.2. Treatment

Treatments of patients included surgery alone (26 cases), preoperative induction chemotherapy plus surgery (3 cases), surgery plus adjuvant radiotherapy and chemotherapy (26 cases), radiotherapy (5 cases), and radiotherapy combined with chemotherapy (3 cases). The surgical approach was expanding lesion resection neck dissection; radiotherapy used three-dimensional conformal radiation therapy or intensity-modulated radiation therapy by external beam linear accelerator, 6MV-X line.

### 2.2. Experimental Method

#### 2.2.1. Preparation of Samples

Pathological slides were stained by H&E and diagnosed by two pathologists. 4 *μ*m thick slides were loaded into 1.5 mL centrifuge tubes for DNA extraction.

#### 2.2.2. PCR Reagents and Primer

QIAamp DNA FFPE Tissue Kit (number 56404) was purchased from Qiagen Company, Germany, to extract tissue DNA. The primer sequences used were HPV16 (F: TCAAAAGCCACTGTGTCCTG; R: CGTGTTCTTGATGATCTGCA); HPV18 (F: TAATAAGGTGCCTGCGGT; R: TCGTTGGAGTCGTTCCTGT).

#### 2.2.3. PCR Amplification

The genomic DNA was extracted from wax-embedded tissue and stored at −20°C. DNA from SiHa cells and HeLa cells was used as HPV16 and HPV18 positive controls and H_2_O was used as negative control. PCR amplification was performed using a temperature gradient and 20 *μ*L reactions which consisted of 2× Premix Ex Taq*™* 10 *μ*L, Forward Primer 0.5 *μ*L, Reverse Primer 0.5 *μ*L, ddH_2_O 7 *μ*L, and genomic DNA template 2 *μ*L. HPV16 amplification parameters were 95°C for 3 min, 95°C for 30 s, an annealing temperature of 55°C for 30 s, 72°C for 30 s, 35 total cycles, and a final extension at 72°C for 5 min. HPV18 amplification parameters were 95°C for 3 min, 95°C for 30 s, an annealing temperature of 57°C for 30 s, 72°C for 30 s, 35 total cycles, and a final extension at 72°C 5 min.

### 2.3. Statistical Methods

In this study, overall survival (OS) was scored as the primary outcome. Death was used as the end point and Kaplan-Meier survival analysis was performed. Univariate analysis was applied to the patient's age, sex, ethnic group, gender, smoking, drinking, M stage, T stage, N stage, tumor differentiation, location, HPV status, and 5-year survival rate. Log-rank testing was also used in the analyses. The COX proportional regression model was used to evaluate possible prognostic factors. The significance level was set at *α* = 0.05.

## 3. Results

### 3.1. HPV16 and HPV18 Infection Rate

In 63 patients with squamous cell carcinoma of the tongue, overall HPV infection rate was 28.6% (18/63). HPV16 infection rate was 23.8% (15/63); HPV18 infection rate was 7.9% (5/63); dual HPV16 and HPV18 infection was found in 2 cases (1 Uighur and 1 Han).

### 3.2. Survival

The follow-up time varied from 8 to 87 months with a median follow-up time of 36 months. Five cases were lost (7.9%). The overall five-year survival rate was 35.6%.

### 3.3. Univariate Analysis

#### 3.3.1. Prognostic Factors

Tumor stage, N stage, and HPV infection were statistically significant in their association with cancer (*P* < 0.05); the age, sex, ethnicity, smoking, drinking, tumor differentiation, and T stage showed no significant association with their prognosis (*P* > 0.05) (Table 1).

#### 3.3.2. The Impact of HPV Infection on the Prognosis of Different Treatment Modalities

*(1) The Impact of HPV Infection on the 3- and 5-Year Survival Rate of Patients with/without Surgery.* Whether or not the patients underwent surgery had no impact on the 3- and 5-year survival rate of HPV-positive or HPV-negative patients (*P* > 0.05) (Table 2).

*(2) The Impact of HPV Infection on the 3- and 5-Year Survival Rate of Patients with/without Radiotherapy.* Patients who had the radiation therapy had a better 3- and 5-year survival rate than those who did not receive radiotherapy (*P* < 0.05) (Table 3).

*(3) The Impact of HPV Infection on the 3- and 5-Year Survival Rate of Patients with/without Chemotherapy.* Patients who received chemotherapy had a better 3-year and 5-year survival rate than those who did not receive chemotherapy (*P* < 0.05) (Table 4).

#### 3.3.3. Multivariate Analysis

COX model multivariate analysis showed that N stage and HPV infection are the significant independent prognostic factors for tongue squamous cell carcinoma (*P* < 0.05). Those patients staging in N2-3 had a 2.538-fold increase in relative risk of death as compared to those in N0-1. HPV-positive patients have a .719-fold reduction in relative risk of death (0.719 times less) compared with patients that are HPV-negative (Table 5).

## 4. Discussion

Xinjiang, located in Western China, comprises 1/6 of the total area of China, equivalent to the size of Iran or to three times the size of France. The religious practices of the different ethnic groups within this region impact their lifestyle especially in regard to strict sanitation and hygiene. The majority of the people residing in this region are also nomadic with no permanent residences. HPV does impact these people and is in general a well known sexually transmitted infection. This makes the patients we selected from a very unique cohort, considering their rural geography and cultural differences, which will give us a different perspective and help enrich our knowledge of tongue squamous cell carcinoma developed from HPV.

Previous studies have indicated that oral squamous cell carcinomas (OSCC) might be correlated with human papilloma virus (HPV) infection. However, the relationship between OSCC in a Chinese population and oral HPV infection is unknown. This is of increasing importance because during last decade there have been significant increases of HPV infections in China. It has been proven that HPV infection is correlated to cervical cancer and anal cancer, but this correlation is still unclear in oropharyngeal cancers. Our study has shown that HPV infection in ethnic minorities living in a closed geographical region elevates the risk of tumorigenesis in an area where sexual transmitted diseases are rare.

It takes years to develop from HPV to cancer and, as the largest oncogene center in Xinjiang, we have accumulated these cases for years. These cases include the major ethnic groups in a relatively restricted geographical area and consists of people who share the same cultural and food preferences. This study provides clinical data to compare with other high HPV prevalence areas and other ethnic groups.

The staging and classification of cancer is an important indicator of clinical treatment and prognosis. In recent years, many studies have shown that HPV is an important and significant factor in the current and future prognosis of patients with head and neck squamous cell carcinoma.

Our results show that HPV-positive patients who are receiving radiation therapy, or chemotherapy, had better 5-year survival rates than HPV-negative patients (*P* < 0.05). This indicates that HPV-positive patients are more sensitive to radiation and chemotherapy. Ang et al. [6] investigated 323 patients with stage III/IV oropharyngeal cancer who underwent radiotherapy. Their results show that HPV-positive patients had a significantly better 3-year survival rate (82.4%) than the HPV-negative group (57.1%). Other studies have shown that sensitivity to radiotherapy and chemotherapy is the key for oropharyngeal cancer prognosis. Hong et al. reported that HPV-DNA positive patients had improved prognosis as compared to HPV-DNA negative patients [7]. More recently, genetic data from Klussmann et al. [8] also showed that HPV-positive tumor cells are more sensitive to radiotherapy and chemotherapy than HPV-negative tumor cells. One possible reason is that the chromosomal aberrations and chromosome doubling in HPV-positive tumors were significantly lower than those in HPV-negative tumors (*P* = 0.030).

Our study including a total of 63 cases of tongue squamous cell carcinoma showed that the overall 5-year survival rate in HPV-positive patients was 47.8%, which is significantly higher than HPV-negative patients (30.3%), (*P* = 0.045). This indicates that the HPV-positive status indicates a better future prognosis than for HPV-negative patients. Lassen et al. [9] reviewed 156 patients with head and neck squamous cell carcinoma and found that 5-year overall survival rate of HPV-positive patients was better than negative ones. Our results showed that HPV-positive patients have reduced relative risk of death by 0.719-fold, consistent with previous research [10, 11].

This study shows that the 5-year overall survival rate in N0-1 and N2-3 stages was 28.2% and 0%, respectively. The univariate statistical analysis showed that patients without lymph node metastasis, or only a single lymph node metastasis, had a better prognosis than multiple lymph node metastasis. Lymph node metastasis is considered the main factor affecting the prognosis of patients [10, 11]. In this study, multivariate statistical analysis also showed that lymph node metastasis is an independent and significant risk factor for the prognosis of tongue squamous cell carcinoma. The multiple lymph node metastasis increased the relative risk of death 2.538-fold as compared to those without lymph node metastasis.

Univariate analysis showed that ethnicity (Han, Uygur, and Kazak) has no impact on prognosis. However, due to the small sample size, this cohort should be expanded and should continue to be followed up.

## 5. Conclusion

To the best of our knowledge, this study includes the biggest sample of tongue squamous cell carcinoma patients from different ethnic groups in Xinjiang, China. The overall infection rate of HPV was 28.6% (18/63) in this cohort; the 5-year survival rate among the HPV-positive patients was 47.8% and 30.3% for HPV-negative patients. The 3-year and 5-year survival rate for HPV-positive patients who received radiotherapy and chemotherapy are better than those who did not receive radiotherapy and chemotherapy. Multivariate analysis showed that N staging and HPV infection are two independent and significant prognostic factors. HPV-positive patients with tongue squamous cell carcinoma are more sensitive to chemotherapy and higher N staging indicates poorer prognosis.

## Figures and Tables

**Table 1:** The prognosis analysis of 63 cases of patients with tongue squamous cell carcinoma.

	Parameters	*n*	5-year survival rate	*χ* ^2^	*P*
Age (yrs)	<60	34	27	0.057	0.812
	≥60	29	48.4		
Gender	Male	25	20.1	2.622	0.105
	Female	38	55.5		
Ethic group	Han	34	36.4	0.007	0.933
	Kazak	10	28.6		
	Uighur	19	49.2		
Smoking	No	44	40.0	0.333	0.564
	Yes	19	21.4		
Alcohol	No	36	46.5	3.063	0.080
	Yes	27	28.7		
staging	I, II	50	45.2	4.975	0.026
	III, IV	13	28.1		
T staging	1, 2	57	47.1	1.045	0.307
	3, 4	6	0		
N staging	0, 1	52	43.8	5.208	0.022
	2, 3	11	31.8		
Differentiation	High	40	57.1	1.054	0.305
	Moderate	23	47.4		
Location	Body	36	25.7	1.733	0.188
	Edge	27	46.3		
HPV	Positive	18	47.8	4.006	0.045
	Negative	45	30.3		

**Table 2:** The impact of HPV infection status on the prognosis of patients undergoing surgery or nonsurgical treatment.

Cases	3-year survival rate		5-year survival rate		*χ* ^2^	*P*
	Surgery	Nonsurgery	Surgery	Nonsurgery		
HPV-positive (*n* = 18)	85.9%	0%	52.7%	0%	0.872	0.350
HPV-negative (*n* = 45)	70.4%	57.1%	42.0%	0%	2.203	0.138

Among 18 patients of HPV-positive patients, 17 patients had surgery, 1 patient did not have surgical treatment; among 45 patients of HPV-negative patients, 38 patients had surgery, 7 patients did not have surgical treatment.

**Table 3:** The impact of HPV infection status on the prognosis of patient receiving radiotherapy or nonradiotherapy.

Cases	3-year survival rate		5-year survival rate		*χ* ^2^	*P*
	Radiotherapy	Nonradiotherapy	Radiotherapy	Nonradiotherapy		
HPV-positive (*n* = 18)	100%	64.3%	66.7%	0%	6.061	0.014
HPV-negative (*n* = 45)	77.0%	59.5%	13.8%	59.5%	0.038	0.846

Among 18 cases of HPV-positive patients, 10 patients received radiotherapy, 8 patients did not receive radiotherapy. Among 45 cases of HPV-negative patients, 24 patients received radiotherapy and 21 patients did not receive radiotherapy.

**Table 4:** The impact of HPV infection on the prognosis of patient with chemotherapy or without chemotherapy.

Cases	3-year survival rate		5-year survival rate		*χ* ^2^	*P*
	Chemotherapy	Nonchemotherapy	Chemotherapy	Nonchemotherapy		
HPV-positive (*n* = 18)	88.9%	65.6%	66.7%	0%	5.743	0.017
HPV-negative (*n* = 45)	58.3%	72.0%	32.4%	24.7%	0.214	0.644

Among 18 cases of HPV-positive patients, 9 patients received chemotherapy, 9 patients did not receive chemotherapy. Among 45 cases of HPV-positive patients, 12 patients received chemotherapy and 21 patients did not receive chemotherapy.

**Table 5:** Multivariate analysis of prognosis in 63 cases of tongue squamous cell carcinoma.

	Coefficient	Standard error	*χ* ^2^	*P*	RR	95.0% CI	
						Lower	Upper
N staging	1.264	0.433	8.509	0.004	3.538	1.514	8.271
HPV infection	−1.268	0.483	6.898	0.009	0.281	0.109	0.725
